# Childhood Threat Is Associated With Lower Resting-State Connectivity Within a Central Visceral Network

**DOI:** 10.3389/fpsyg.2022.805049

**Published:** 2022-03-03

**Authors:** Layla Banihashemi, Christine W. Peng, Anusha Rangarajan, Helmet T. Karim, Meredith L. Wallace, Brandon M. Sibbach, Jaspreet Singh, Mark M. Stinley, Anne Germain, Howard J. Aizenstein

**Affiliations:** ^1^Department of Psychiatry, University of Pittsburgh, Pittsburgh, PA, United States; ^2^Department of Bioengineering, University of Pittsburgh, Pittsburgh, PA, United States; ^3^Department of Statistics, University of Pittsburgh, Pittsburgh, PA, United States; ^4^Department of Neuroscience, University of Pittsburgh, Pittsburgh, PA, United States; ^5^Department of Psychology, University of Pittsburgh, Pittsburgh, PA, United States

**Keywords:** childhood trauma, subgenual anterior cingulate cortex, amygdala, bed nucleus of stria terminalis, extended amygdala, hypothalamus, affective disorders

## Abstract

Childhood adversity is associated with altered or dysregulated stress reactivity; these altered patterns of physiological functioning persist into adulthood. Evidence from both preclinical animal models and human neuroimaging studies indicates that early life experience differentially influences stressor-evoked activity within central visceral neural circuits proximally involved in the control of stress responses, including the subgenual anterior cingulate cortex (sgACC), paraventricular nucleus of the hypothalamus (PVN), bed nucleus of the stria terminalis (BNST) and amygdala. However, the relationship between childhood adversity and the resting-state connectivity of this central visceral network remains unclear. To this end, we examined relationships between childhood threat and childhood socioeconomic deprivation, the resting-state connectivity between our regions of interest (ROIs), and affective symptom severity and diagnoses. We recruited a transdiagnostic sample of young adult males and females (*n* = 100; mean age = 27.28, *SD* = 3.99; 59 females) with a full distribution of maltreatment history and symptom severity across multiple affective disorders. Resting-state data were acquired using a 7.2-min functional magnetic resonance imaging (fMRI) sequence; noted ROIs were applied as masks to determine ROI-to-ROI connectivity. Threat was determined by measures of childhood traumatic events and abuse. Socioeconomic deprivation (SED) was determined by a measure of childhood socioeconomic status (parental education level). Covarying for age, race and sex, greater childhood threat was significantly associated with lower BNST-PVN, amygdala-sgACC and PVN-sgACC connectivity. No significant relationships were found between SED and resting-state connectivity. BNST-PVN connectivity was associated with the number of lifetime affective diagnoses. Exposure to threat during early development may entrain altered patterns of resting-state connectivity between these stress-related ROIs in ways that contribute to dysregulated neural and physiological responses to stress and subsequent affective psychopathology.

## Introduction

Due to its high prevalence (Hillis et al., 2016; Merrick et al., 2018; Cuartas et al., 2019) and importance as a predictor of affective risk, childhood adversity is at the forefront of psychiatry’s public health burden (Sara and Lappin, 2017). One sensitivity analysis of global past year violence against children found that a minimum of 1.4 out of 2 billion children aged 2–17 experienced physical, sexual or emotional violence (Hillis et al., 2016). Further, the COVID-19 pandemic has exacerbated systemic challenges, increasing children’s risk of violence exposure (M’jid, 2020; Pereda and Díaz-Faes, 2020). Childhood adversity is a risk factor for and prospective predictor of greater affective symptoms and disorders (Danese et al., 2009; Nanni et al., 2012; Baldwin et al., 2021; Mayer et al., 2021; Russotti et al., 2021). Thus, greater mechanistic understanding of childhood adversity-related neural and physiological differences is necessary to mitigate these risks and guide treatment efforts.

Childhood adversity is associated with dysregulated (heightened or diminished) stress reactivity in childhood and later in life (Al’Absi et al., 2021), with alterations in both neuroendocrine and autonomic physiology and stress reactivity (Heim et al., 2000, 2001; Chen et al., 2004; Koopman et al., 2004; Carpenter et al., 2007, 2011; Gunnar et al., 2009; Lovallo et al., 2011; Hackman et al., 2012). There is evidence to suggest that there may be differential influences of childhood adversity dimensions, threat (e.g., abuse, traumatic events) and deprivation (e.g., neglect, socioeconomic deprivation, institutional rearing) (McLaughlin et al., 2014; Sheridan and McLaughlin, 2014) on stress reactivity, with threat blunting (Carpenter et al., 2007, 2011; Doom et al., 2014; Peckins et al., 2015; Bernard et al., 2017) and deprivation (i.e., low socioeconomic status, SES) heightening reactivity (Lupien et al., 2001; Cohen et al., 2006; Chen et al., 2009; Lê-Scherban et al., 2018). Despite evidence linking childhood threat and deprivation to altered physiological stress systems, how childhood adversity shapes specific, proximally stress-responsive neural circuits remains unclear.

**FIGURE 1:**
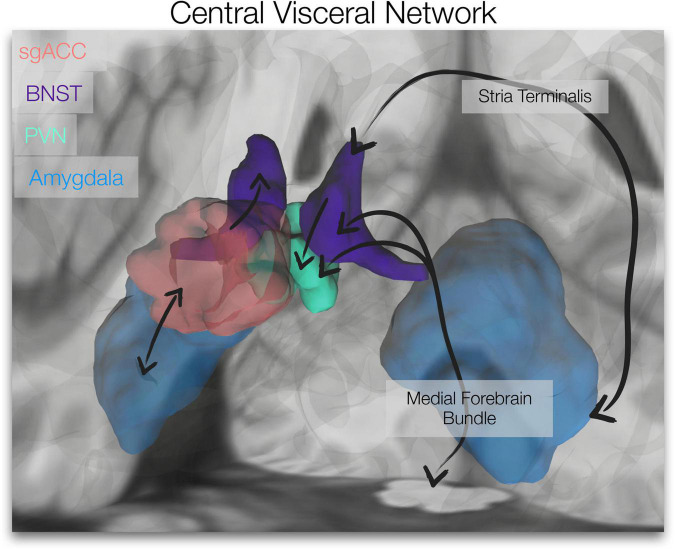
Central Visceral Network. The brain image depicts the regions of interest utilized in our resting-state analyses, the subgenual anterior cingulate cortex (sgACC, red), bed nucleus of the stria terminalis (BNST, violet), paraventricular nucleus of the hypothalamus (PVN, green) and amygdala (blue). Simplified connections are depicted (black arrows; not specific with respect to subnuclei). Ascending viscerosensory and descending preautonomic/visceromotor pathways course through the medial forebrain bundle (BNST and PVN connections depicted). Amygdala, BNST and PVN are connected via the stria terminalis. BNST projections to PVN and sgACC innervation of the BNST are displayed, as well as reciprocal connections between the sgACC and amygdala. The amygdalofugal pathway is also integral to this network (not depicted here). (Unilateral representations are shown).

The subgenual anterior cingulate cortex (sgACC), paraventricular nucleus of the hypothalamus (PVN), bed nucleus of the stria terminalis (BNST) and amygdala form a stress-responsive, central visceral network. These limbic forebrain and hypothalamic regions are central to reciprocal descending preautonomic/visceromotor and ascending viscerosensory (i.e., central visceral) pathways (see Figure 1) that control and modulate autonomic outflow and neuroendocrine function (Banihashemi and Rinaman, 2006; Card and Sved, 2011; Rinaman et al., 2011). Further, connections between these regions are important for stress regulation; the PVN, a gateway of hypothalamic-pituitary-adrenal (HPA)/neuroendocrine and autonomic regulation (Luiten et al., 1985; Herman et al., 2002), is directly innervated and influenced by the BNST (Dong et al., 2001b; Povysheva et al., 2021). With little to no innervation of the PVN (Freedman et al., 2000), the sgACC/Brodmann area 25 may access the PVN via direct connections to the BNST (Freedman et al., 2000; Vertes, 2004). The amygdala also has reciprocal connections to the sgACC (Heilbronner and Haber, 2014; Oler and Fudge, 2019; Sharma et al., 2019) and BNST (Dong et al., 2001a; Bienkowski et al., 2013; deCampo and Fudge, 2013; Oler et al., 2017; Figure 1). Further, the intrinsic functional connectivity of the extended amygdala (BNST/amygdala) appears to align with known anatomical connectivity; Tillman et al. (2018) showed that compared to the central nucleus of the amygdala, the BNST displayed stronger coupling with anterior cortical areas, including ventromedial prefrontal cortex/sgACC and brainstem/dorsal periaqueductal gray. This network (along with its central visceral connections) is involved in affective processes (e.g., emotional memory, threat responses, fear and anxiety) (Fendt et al., 2005; Schweimer et al., 2005; Somerville et al., 2010; Avery et al., 2014, 2016; Herrmann et al., 2016), implicated in psychopathology (e.g., depression, anxiety, trauma-related disorders) (Thayer and Lane, 2000; Gotlib et al., 2005; Drevets et al., 2008b; Lebow and Chen, 2016; Clauss, 2019; Clauss et al., 2019), and targeted for affective disorder treatments (e.g., deep brain stimulation for depression and obsessive compulsive disorder) (Johansen-Berg et al., 2008; Gutman et al., 2009; Drobisz and Damborská, 2018; Fitzgerald et al., 2018; Mosley et al., 2021).

Previous research in preclinical animal models (Banihashemi and Rinaman, 2010; Banihashemi et al., 2011) and human neuroimaging (Banihashemi et al., 2015) indicates that early life experience differentially influences stressor-evoked activity within this visceral, stress-responsive network. In physically and mentally healthy adults, childhood threat (i.e., physical abuse) is significantly associated with greater stressor-evoked activity across this central visceral, limbic forebrain-hypothalamic network (Banihashemi et al., 2015). Yet, how childhood adversity may influence resting-state connectivity of this central visceral network is virtually unknown.

Studies focused on childhood adversity and resting-state connectivity have spanned development and have primarily focused on amygdala-related or large-scale network connectivity (Teicher et al., 2020). In youth, findings tend to indicate that greater childhood maltreatment or trauma exposure is associated with greater amygdala-related connectivity (amygdala-sgACC cortex, amygdala-hippocampus, amygdala-salience network) (Marusak et al., 2015; Thomason et al., 2015; Rakesh et al., 2021b), while late adolescents/adults tend to display an opposing trend with greater maltreatment associated with less amygdala-related connectivity (amygdala-sgACC, amygdala-ventromedial prefrontal cortex, amygdala-orbitofrontal cortex/insula/hippocampus, amygdala-cuneus/precuneus) (Werff et al., 2012; Herringa et al., 2013; Souza-Queiroz et al., 2016). Rabellino et al. (2018) investigated BNST resting-state connectivity in PTSD and its dissociative subtype; however, they found no significant relationship between childhood maltreatment and BNST resting-state connectivity in subsequent analyses. To our knowledge, no studies of childhood adversity have focused exclusively on the resting-state connectivity of the central visceral network described. Thus, the current study is unique in examining this network in a transdiagnostic sample recruited specifically to represent a spectrum of severity across self-reported childhood physical abuse.

Our recent work discovered opposing relationships of childhood threat (both abuse and traumatic events) and socioeconomic deprivation with white matter structural integrity (i.e., generalized fractional anisotropy, gFA) of the stria terminalis (Banihashemi et al., 2021), which connects several of our regions of interest (ROIs) (caudomedial amygdala, BNST and hypothalamus) (Rafal et al., 2015; Oler et al., 2017; Weiss et al., 2021). Greater threat was associated with less, while greater deprivation was associated with greater, stria terminalis gFA. Thus, we hypothesized that threat and deprivation would have congruent, opposing effects on resting-state connectivity within this central visceral network (i.e., threat would be associated with lower, while deprivation would be associated with greater, resting-state connectivity). To this end, our goal was to examine differential relationships of threat and deprivation with resting-state connectivity between our central visceral ROIs, as well as to examine how their functional connectivity may contribute to affective symptoms or disorders. These findings may contribute to a greater understanding of how childhood-adversity shapes this central visceral network at rest, how these resting state dynamics may prime functional activity in response to emotionally salient stimuli and how this network may contribute to childhood adversity-related differences in affective symptoms and disorders.

## Materials and Methods

### Participants

Participants were recruited from Allegheny County, Pittsburgh, PA, United States, using various methods (e.g., referrals from other research studies, and online and bus advertisements). Of the 1,020 contacts made, 111 (18.5%) underwent informed consent and were enrolled in the study. Of those consented, 100 (90%) participants completed study procedures. Participants were 59 female and 41 male young adults (*n* = 100, mean age = 27.28, *SD* = 3.99). Of the 100 participants that completed the study, 45% self-reported their race as White, 36% as Black or African American, 13% as Asian, 4% as multiracial, and 2% as biracial. Within this sample, 7 individuals reported their ethnicity as Hispanic or Latin American. All participants provided informed consent after receiving an explanation of study protocols and were examined with the approval of the University of Pittsburgh Institutional Review Board.

Exclusion criteria were: Magnetic resonance imaging (MRI) contraindications (e.g., claustrophobia, metal in the body, severe visual or auditory impairment), pregnancy, left-handedness, cardiovascular disease and diabetes, neurological disorders (including seizure disorders, migraine disorder, traumatic brain injury, or neurodegenerative disorders), psychotropic medications or any medications affecting cardiovascular or neural function, suicidality or marked functional impairment, and current psychiatric disorders (bipolar, psychotic disorders, substance abuse or dependence) except for depression, anxiety or trauma-related disorders.

Individuals were also screened using the five childhood physical abuse items from the Childhood Trauma Questionnaire (CTQ) with the goal of achieving an even distribution of participants across four physical abuse severity classifications as defined by CTQ guidelines (Bernstein et al., 1994). The following distribution of physical abuse severity was achieved in the final sample (*n* = 100): 29% None-Minimal, 23% Low-Moderate, 21% Moderate-Severe, 27% Severe-Extreme. A balanced distribution was also achieved across childhood SES (as assessed by maximum parental education level), 31% Low (GED – some college, no degree), 34% Middle (Associate or Bachelor’s), and 35% High (Master’s or Doctorate). All participants had at least one parent with a GED or higher education level.

### Study Protocol and Measures

The study comprised two visits completed within one month (mean number of days between visits: 14.39 ± 10.96), an intake visit followed by an MRI scan visit at the University of Pittsburgh Magnetic Resonance Research Center. At the first visit, eligibility was determined using medical history, two-week medication history, current substance use and traumatic brain injury inventories. Participants were excluded if deemed ineligible by these assessments.

#### Childhood Threat

Childhood Threat was assessed with the Childhood Trauma Questionnaire (CTQ) and the Trauma History Questionnaire (THQ). The CTQ is a 28-item Likert-type scale that examines five subscales of maltreatment: physical, emotional and sexual abuse, and physical and emotional neglect (Bernstein et al., 1994). Each subscale contains five items with scores ranging from 1-Never to 5-Very Often True. A sum of the three abuse subscales represented our CTQ Threat variable (with 15 indicating no abuse and 75 indicating extreme abuse). (A sum of the two neglect subscales represented our CTQ Deprivation variable, which was used in secondary analyses. See section “Variable Selection”).

The THQ is a 24-item questionnaire that assesses the occurrence of traumatic events throughout the life course (Stamm, 1996). An adapted version of the THQ was used in which participants responded yes or no to indicate whether a particular event occurred and then selected the relevant age range(s): age 0–11, age 12–17, and age > 18 (Insana et al., 2012). Traumatic events included experiences with crime, environmental disasters, injury or death, as well as physical or sexual abuse. THQ 0-11 was used as a primary measure of childhood threat and THQ > 18 was used as a covariate (see sections “Variable Selection” and “Data Analysis”).

#### Childhood Socioeconomic Deprivation

A sociodemographic inventory was used to assess childhood and adulthood SES. Maximum parental education level was used to determine childhood SES; the participants’ own educational level determined adulthood SES. Both were presented as a 9-point education level scale (0 - No high school diploma, 1 – GED, 2 – High school diploma, 3 – Technical training, 4 – Some college, no degree, 5 – Associate degree, 6 – Bachelor’s degree, 7 – Master’s degree, 8 – MD/PhD/JD/PharmD). The Childhood Deprivation construct encompasses low SES, socioeconomic disadvantage or neighborhood deprivation (McLaughlin et al., 2014; Webb et al., 2017; Berti and Pivetti, 2019; Morris et al., 2019). Further, education level is often used as a measure of SES and is associated with mental health inequalities (Reiss, 2013), physiological stress (Ursache et al., 2017) and physical health, especially cardiovascular disease risk (Winkleby et al., 1992). Thus, we used maximum parental education level (reverse coded) as our primary measure of childhood socioeconomic deprivation (SED). Adulthood SES was used as a covariate.

#### Negative Life Events

The 24-item Life Events List assesses significant life events experienced by the participant within the past 12 months (e.g., unemployment, separation or divorce, serious illness, death of someone close) (Cohen et al., 1991). Participants indicate whether or not they have experienced an event in the past year with follow up questions assessing valence and/or details if endorsed. This inventory was used to assess the total number of negative life events, which was used as a covariate.

#### Affective Symptom Severity

Depression and post-traumatic stress symptom severity were assessed using the Beck’s Depression Inventory (BDI-II) and the PTSD Checklist - Civilian Version (PCL-C), respectively. The BDI-II is a 21-item questionnaire that assesses the presence and severity of depression within the past two weeks; it probes whether participants have experienced a thought or behavior related to depressive symptoms on a scale of 0 to 3, with scores > 20 considered moderate-to-severe (Beck et al., 1996). The PCL-C is a 20-item measure that reliably assesses post-traumatic stress symptom severity in the last month on a 5-point Likert scale ranging from not at all (Cuartas et al., 2019) to extremely (M’jid, 2020), with scores > 30 considered moderate-to-severe. It includes assessment of re-experiencing, avoidance and arousal symptoms, as well as negative cognitions (Wilkins et al., 2011).

#### Diagnostic Assessment

Psychiatric diagnoses of mood, anxiety or trauma-related disorders were evaluated and confirmed via in-person interview using the Structured Clinical Interview for DSM-IV Axis I Disorders by a trained interviewer. Of the 100 participants who completed the study, 29% were healthy (had no history of the affective disorders evaluated), whereas 71% had a history of affective diagnosis (47 participants had one or more current affective diagnoses). Of those with a diagnostic history, 30 had a trauma-related disorder, 24 had a depressive disorder and 17 had an anxiety disorder, as their primary lifetime diagnosis. Posttraumatic stress disorder was the most frequent diagnosis (30% of the sample) followed by major depressive disorder (15% of the sample). Further, 37% had comorbid lifetime mood and anxiety/trauma-related disorders.

#### Sample Characterization

Participants also completed questionnaires to characterize the sample, including the Perceived Stress Scale (PSS, 10-item version) to assess frequency of stress-related feelings (Cohen et al., 1983), the State Trait Anxiety Inventory (STAI-Y2) to assess presence and severity of trait anxiety (Spielberger et al., 1983) and the NEO Five-Factor Inventory-3 (NEO-FFI-3, 60 items) to assess personality (McCrae and Costa, 2007). See Supplementary Table S1 for Participant Characteristics.

#### Magnetic Resonance Imaging Protocol and Data Acquisition

Magnetic resonance imaging data were collected on a 3-Tesla Trio TIM whole-body MRI scanner (Siemens, Erlangen, Germany), equipped with a 32-channel head coil. Prior to the resting-state sequence, participants were instructed to “gaze at the fixation cross and rest” and reminded to remain as still as possible. A custom, localized shimming procedure was implemented that extended from the bottom-most slice to the ventral aspect of the corpus callosum. Resting-state functional MRI data were acquired using a 7.2-min, T2*-weighted gradient-echo echoplanar imaging (EPI) sequence (TR = 2000 ms, TE = 29 ms, flip angle = 65°, slices = 22, Multiband Factor = 3, FoV = 220 × 220 mm^2^, voxel size = 2 × 2 × 2.0 mm^3^). The FOV was angled 15–20° to ensure visualization of our ROIs. For registration purposes, anatomical images were acquired using a 4.8-min T1-weighted sagittal MPRAGE sequence (TR = 1500 ms, TE = 3.19 ms, flip angle = 8°, 176 slices, FoV = 256 × 256 mm^2^, voxel size = 1 × 1 × 1.0 mm^3^). The resting-state sequence followed the MPRAGE acquisition, which was the first sequence in the protocol. Additional sequences were collected during the MR (not reported here) with a total duration of approximately 50–55 min.

#### Resting-State Functional Magnetic Resonance Imaging Preprocessing and Analysis

Resting state fMRI data were preprocessed using Statistical Parametric Mapping software (SPM12^1^). Motion correction was applied through realignment of each blood-oxygen-level dependent (BOLD) image to the mean reference image. The structural image was then co-registered to the mean functional image. Segmentation was performed on the structural image using probability maps for six tissue classes, generating a deformation field that was then applied to the functional images during normalization of all images to standard Montreal Neurological Institute (MNI) space (2 mm isotropic resolution). Smoothing was applied to functional images using a 4 mm full-width-at-half-maximum Gaussian kernel.

Resting-state connectivity analyses were performed using standard SPM-based functions (in-house MATLAB code was used to wrap these functions). Translation (mm) and rotation (deg) was assessed for each participant; motion was low across the sample of 100 participants (Translation: mean = 1.13 mm, *SD* = 0.37; Rotation: mean = 0.86 degrees, *SD* = 0.73). Our threshold for maximum translation was 3 mm of motion and none exceeded this. Motion artifact reduction was applied to smoothed functional images using the SPM BrainWavelet Toolbox wavelet despiking methods to identify and filter spike artifacts. A principal component analysis was performed by extracting five eigenvariates of the BOLD signal principal time series from the white matter and cerebrospinal fluid simultaneously using singular value decomposition. Using multiple linear regression, the time series at each voxel was adjusted by applying these tissue components and the raw values of the six motion parameters (not their derivatives) from preprocessing as covariates. The residual time series was extracted from each voxel and we used a series of cosines to model the band pass Butterworth filter (0.008–0.15 Hz), which was applied on the residuals.

#### Region of Interest-to-Region of Interest Analyses

The sgACC, BNST and PVN ROI masks were created using manual segmentation with MRIcron on the ch2better template. The BNST and PVN ROIs were based on the Atlas of the Human Brain (Mai et al., 2008) [BNST: plates 18 (Talairach reference systems, *y* = −2.7 mm) through 24 (*y* = + 2.7 mm); PVN: plates 20 (*y* = −1.3 mm) through 28 (*y* = + 8.0 mm)]. The sgACC ROI was based on its depiction in Cingulate Neurobiology and Disease (Vogt, 2009). These ROIs were described initially (Banihashemi et al., 2015) and utilized/reported elsewhere (Andreescu et al., 2015a,b; Price et al., 2018; Wu et al., 2019). The amygdala ROI was created from the SPM Anatomy toolbox using the 50% probabilistic map (Amunts et al., 2005; Eickhoff et al., 2005). Each ROI was applied as a mask on the covariate-processed functional data. Using principal component analysis, we extracted the first eigenvariate within each ROI for each subject. The correlation (Pearson) between the eigenvariates for each ROI-to-ROI pair was calculated to determine connectivity between the two regions.

### Variable Selection

Our rationale for childhood threat and deprivation variable selection has been described previously (Banihashemi et al., 2021). Briefly, preliminary data analyses from the current sample demonstrated that our childhood threat and deprivation variables were correlated (Table 1), however, among deprivation measures, socioeconomic deprivation [SED, maximum parental education level (reverse coded, such that higher values reflected greater deprivation)] was the least correlated with the threat measures (Pearson *r* = 0.175 to 0.411, Table 1). CTQ Threat (abuse) and CTQ Deprivation (neglect) were strongly correlated (*r* = 0.769). As such, CTQ Deprivation was considered only in exploratory analyses (data not shown) and SED was used as the primary measure of childhood deprivation. As early childhood experiences are critical for brain development (Tottenham and Sheridan, 2010), our primary analyses of trauma utilized early traumatic events (THQ 0-11); exploratory analyses examining later traumatic events (THQ 12-17) are included in the Supplementary Material. Because CTQ Threat and THQ 0-11 are highly correlated (*r* = 0.535), these threat measures were considered in separate models, allowing separate examination of broader traumatic events (THQ 0-11) and abuse (CTQ Threat). Because threat co-occurs with SED (and these constructs are not independent from one another), we examined the additive effects (Fahrmeir et al., 2013) of threat and deprivation similar to Lawson et al. (2017).

**TABLE 1:** Pearson correlations between childhood threat and deprivation measures (*n* = 100).

		CTQ Threat	THQ 0-11	THQ 12-17	CTQ deprivation	SED
CTQ Threat (abuse)	Pearson *r*	–				
	*p* (2-tailed)					
THQ 0-11	Pearson *r*	0.535**	–			
	*p* (2-tailed)	0.000				
THQ 12-17	Pearson *r*	0.580**	0.539**	–		
	*p* (2-tailed)	0.000	0.000			
CTQ Deprivation (neglect)	Pearson *r*	0.769**	0.462**	0.534**	–	
	*p* (2-tailed)	0.000	0.000	0.000		
Socioeconomic Deprivation (SED)	Pearson *r*	0.411**	0.175	0.337**	0.404**	–
	*p* (2-tailed)	0.000	0.081	0.001	0.000	

**Correlation is significant at the 0.01 level (2-tailed).

### Data Analysis

#### Childhood Adversity and Resting-State Connectivity

We examined whether childhood threat and SED variables were associated with resting-state connectivity between our ROIs; six resting-state ROI-to-ROI connections were examined (Amygdala-BNST, Amygdala-PVN, Amygdala-sgACC, BNST-PVN, BNST-sgACC and PVN-sgACC). All hierarchical linear regression models covaried for age, race and sex in Step 1 and examined the additive effects of childhood threat and SED together in Step 2 (i.e., Model 1: early traumatic events and SED, and Model 2: abuse and SED). We also evaluated whether our findings remained after multiple comparison correction (FDR < 0.05, for six tests, one for each ROI-to-ROI connection) (Benjamini and Hochberg, 1995) and after adjusting for adulthood trauma (all traumatic events occurring after age 18, THQ > 18), adulthood SES (education level) and negative life events within the past year (Life Events List); these variables were entered together in Step 3. Where a significant relationship was found between abuse (CTQ Threat) and resting-state connectivity, *post hoc* analyses were performed substituting each abuse subscale in the model to examine which type of abuse may have been driving the relationship.

#### Resting-State Connectivity and Affective Symptoms/Disorders

Where a significant relationship was found between childhood threat and resting-state connectivity between our ROIs, we also examined whether ROI-to-ROI connectivity was associated with depressive or post-traumatic stress symptom severity or the number of lifetime diagnoses. Hierarchical regression models covaried for age, race and sex in Step 1 and examined the effect of ROI-to-ROI connectivity in Step 2 in separate models. We also evaluated whether our findings remained after multiple comparison correction (FDR < 0.05, for three tests, one for each measure of affect) and after adjusting for adulthood trauma (THQ > 18), adulthood SES (education level) and negative life events within the past year (Life Events List); these variables were entered together in Step 3.

## Results

### Childhood Threat, Deprivation and Central Visceral Network Resting-State Connectivity

#### Early Traumatic Events (Trauma History Questionnaire, Age 0–11)

**FIGURE 2:**
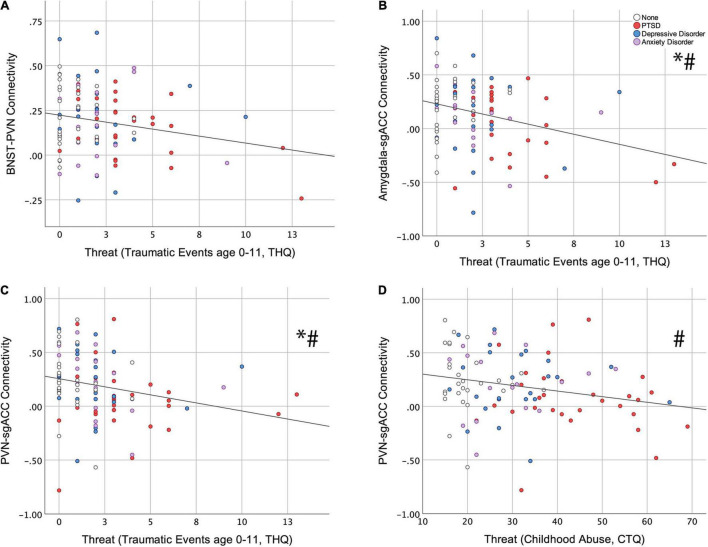
Relationships between childhood threat and central visceral network resting-state connectivity. Hierarchical linear regression was used to examine associations between early traumatic events (Trauma History Questionnaire, THQ, age 0–11) and **(A)** BNST-PVN (ß = −0.224; *p* = 0.033), **(B)** Amygdala-sgACC (ß = −0.312; *p* = 0.003), and **(C)** PVN-sgACC (ß = −0.264; *p* = 0.008) connectivity. **(D)** The association between childhood abuse (Childhood Trauma Questionnaire, CTQ) and PVN-sgACC (ß = −0.258; *p* = 0.018) connectivity. * Indicates survival of FDR correction (0.05) for six tests; # indicates that the childhood threat variable remained significant with the addition of adulthood covariates (adulthood trauma, socioeconomic status and negative life events) to the model. (BNST, bed nucleus of the stria terminalis; PVN, paraventricular nucleus of the hypothalamus; sgACC, subgenual anterior cingulate cortex).

Of the six resting-state ROI-to-ROI connections examined, THQ 0-11 had a significant, negative association with BNST-PVN (ß = −0.224; *p* = 0.033, Figure 2A), Amygdala-sgACC (ß = −0.312; *p* = 0.003, Figure 2B) and PVN-sgACC connectivity (ß = −0.264; *p* = 0.008, Figure 2C) (standardized ß values reported throughout; Table 2). Among these, relationships between THQ 0-11 and Amygdala-sgACC (adjusted *p* = 0.018) and PVN-sgACC (adjusted *p* = 0.024) survived multiple comparison correction, and both relationships remained significant when adulthood trauma (age > 18), adulthood SES and negative life events were added to the model [Amygdala-sgACC (ß = −0.328; *p* = 0.005); PVN-sgACC (ß = −0.241; *p* = 0.030) (Table 2)]. Socioeconomic deprivation (SED, maximum parental education level reverse coded) did not have a significant association with any ROI-to-ROI connection examined.

**TABLE 2:** Hierarchical linear regression results: childhood threat (early traumatic events, age 0–11) and central visceral network resting-state connectivity.

		BNST-PVN			Amygdala-sgACC			PVN-sgACC		
Step	Variable	St. Beta	*t*	*p*	St. Beta	*t*	*p*	St. Beta	*t*	*p*
1	Age	–0.107	–1.050	0.296	–0.036	–0.358	0.721	0.113	1.166	0.247
	Race	0.092	0.910	0.365	–0.177	–1.750	0.083	–0.038	–0.388	0.699
	Sex	0.096	0.943	0.348	0.007	0.068	0.946	0.301	3.111	**0.002**
2	Age	–0.085	–0.809	0.421	–0.007	–0.069	0.945	0.152	1.529	0.130
	Race	0.135	1.330	0.187	–0.117	–1.184	0.240	0.010	0.104	0.917
	Sex	0.097	0.961	0.339	0.009	0.093	0.926	0.295	3.102	**0.003**
	**THQ 0-11**	–0.224	–2.170	**0**.**033**	–0.312	–3.103	0.003*	–0.264	–2.707	0.008*
	Socioeconomic Deprivation	0.084	0.800	0.425	0.119	1.161	0.249	0.049	0.492	0.624
3	Age	0.008	0.067	0.947	–0.025	–0.210	0.834	0.161	1.417	0.160
	Race	0.121	1.183	0.240	–0.119	–1.170	0.245	0.003	0.026	0.979
	Sex	0.073	0.686	0.495	0.015	0.143	0.886	0.257	2.511	**0.014**
	**THQ 0-11**	–0.151	–1.312	0.193	–0.328	–2.887	0.005*	–0.241	–2.201	**0.030**
	Socioeconomic Deprivation	0.074	0.639	0.524	0.108	0.944	0.348	0.053	0.487	0.628
	THQ > 18	–0.182	–1.385	0.169	0.039	0.299	0.766	0.048	0.384	0.702
	Adulthood SES	–0.139	–1.301	0.197	–0.006	–0.057	0.955	–0.037	–0.364	0.717
	Negative Life Events	–0.047	–0.387	0.699	0.013	0.103	0.918	–0.134	–1.143	0.256

*Bold values indicate significance at p < 0.05; an asterisk indicates survival of FDR correction (0.05) for six tests.*

An examination of potential outliers is reported in the Supplementary Material [Results, Early Traumatic Events (THQ, age 0–11, Supplementary Tables S2–S4)]. When removing the largest THQ 0-11 value, the amygdala-sgACC and PVN-sgACC results remain significant, continue to survive multiple comparison correction and remain significant with the additional adulthood covariates.

#### Abuse (Childhood Trauma Questionnaire Threat)

CTQ Threat had a significant, negative association with only one resting-state ROI-to-ROI connection, PVN-sgACC connectivity (ß = −0.258; *p* = 0.018, Figure 2D and Table 3). This finding did not survive multiple comparison correction (adjusted *p* = 0.108); however, it did remain significant (ß = −0.228; *p* = 0.043) with the additional adulthood covariates (trauma, SES and negative life events) (Table 3). SED did not have a significant association with any ROI-to-ROI connection examined.

**TABLE 3:** Hierarchical linear regression results: childhood threat (abuse) and central visceral network resting-state connectivity.

		PVN-sgACC		
Step	Variable	St. Beta	*t*	*p*
1	Age	0.113	1.166	0.247
	Race	–0.038	–0.388	0.699
	Sex	0.301	3.111	**0.002**
2	Age	0.150	1.494	0.139
	Race	0.004	0.043	0.966
	Sex	0.262	2.696	**0.008**
	**CTQ Threat**	–0.258	–2.398	**0.018**
	Socioeconomic Deprivation	0.105	0.986	0.327
3	Age	0.189	1.660	0.100
	Race	0.003	0.028	0.978
	Sex	0.214	2.081	**0.040**
	**CTQ Threat**	–0.228	–2.057	**0.043**
	Socioeconomic Deprivation	0.124	1.070	0.287
	THQ > 18	–0.004	–0.033	0.974
	Adulthood SES	–0.029	–0.284	0.777
	Negative Life Events	–0.166	–1.448	0.151

*Bold values indicate significance at p < 0.05.*

*Post hoc* analyses examining CTQ Threat subscales (physical, emotional and sexual abuse) revealed that physical (ß = −0.255; *p* = 0.023, Supplementary Table S5) and sexual (ß = −0.250; *p* = 0.014, Supplementary Table S6) abuse were each negatively associated with PVN-sgACC connectivity. Both findings survived multiple comparison correction for 3 tests (one for each abuse type) [physical abuse (adjusted *p* = 0.035); sexual abuse (adjusted *p* = 0.042)]; however, only sexual abuse remained significant (ß = −0.248; *p* = 0.015) with the additional adulthood covariates (Supplementary Tables S5, S6).

#### Exploratory Analyses

Exploratory analyses examining later traumatic events (THQ 12-17) are included in Supplementary Table S7.

When CTQ Deprivation (Neglect) was substituted for SED in the THQ 0-11 or CTQ Threat models, no significant associations of CTQ Deprivation with any ROI-to-ROI connection were observed (data not shown).

### Central Visceral Network Resting-State Connectivity and Affective Symptoms or Diagnoses

Of the three ROI-to-ROI connections that showed an association with childhood threat (early traumatic events and/or childhood abuse) (BNST-PVN, Amygdala-sgACC, PVN-sgACC), only BNST-PVN connectivity was associated with affective (depressive or post-traumatic stress) symptoms or diagnoses. BNST-PVN connectivity was negatively associated with depressive symptoms (ß = −0.138; *p* = 0.169, non-significant), post-traumatic stress symptoms (ß = −0.202; *p* = 0.045) and the number of lifetime affective diagnoses (ß = −0.236; *p* = 0.011, Table 4). The relationship between BNST-PVN connectivity and the number of lifetime diagnoses survived multiple comparison correction (adjusted *p* = 0.033) and remained significant when adulthood trauma, adulthood SES and negative life events were added to the model (ß = −0.191; *p* = 0.032, Table 4). Relationships between childhood adversity and affective symptoms/diagnoses are in Supplementary Table S8.

**TABLE 4:** Hierarchical linear regression results: central visceral network resting-state connectivity and affective symptoms or diagnoses.

		BDI-II			PCL-C			Lifetime Diagnoses		
Step	Variable	St. Beta	*t*	*p*	St. Beta	*t*	*p*	St. Beta	*t*	*p*
1	Age	0.223	2.238	**0.028**	0.190	1.885	0.062	0.337	3.618	**0.000**
	Sex	–0.148	–1.486	0.141	–0.117	–1.167	0.246	–0.296	–3.189	**0.002**
	Race	–0.004	–0.042	0.967	0.029	0.286	0.776	–0.042	–0.450	0.653
2	Age	0.208	2.088	**0.039**	0.168	1.688	0.095	0.311	3.427	**0.001**
	Sex	–0.134	–1.354	0.179	–0.098	–0.986	0.327	–0.273	–3.019	**0.003**
	Race	0.009	0.086	0.932	0.047	0.477	0.634	–0.020	–0.221	0.826
	**BNST-PVN Connectivity**	–0.138	–1.385	0.169	–0.202	–2.032	**0.045**	–0.236	–2.603	0.011*
3	Age	0.039	0.352	0.725	–0.049	–0.451	0.653	0.131	1.320	0.190
	Sex	–0.029	–0.288	0.774	0.031	0.314	0.754	–0.170	–1.885	0.063
	Race	–0.021	–0.218	0.828	0.016	0.176	0.861	–0.057	–0.665	0.508
	**BNST-PVN Connectivity**	–0.088	–0.898	0.372	–0.128	–1.347	0.181	–0.191	–2.177	**0.032**
	THQ > 18	0.186	1.560	0.122	0.247	2.149	**0.034**	0.220	2.078	**0.040**
	Adulthood SES	–0.048	–0.491	0.624	0.014	0.145	0.885	–0.101	–1.169	0.245
	Negative Life Events	0.253	2.264	**0.026**	0.320	2.973	**0.004**	0.227	2.285	**0.025**

*Bold values indicate significance at p < 0.05; an asterisk indicates survival of FDR correction (0.05) for six tests.*

## Discussion

An expanding literature has linked childhood adversity to neural measures of brain structure, (e.g., gray matter volume, van Harmelen et al., 2010; Walsh et al., 2014) and white matter structural integrity (Choi et al., 2012; Hanson et al., 2013, 2015) and function [e.g., emotion or threat processing (Fani et al., 2010; McCrory et al., 2011, 2013; van Harmelen et al., 2012) and resting-state connectivity] (Teicher et al., 2016; Ho and King, 2021). Further, threat and deprivation dimensions of childhood adversity may have different neural correlates (McLaughlin et al., 2014; Sheridan and McLaughlin, 2014) and/or influence the same neural circuits in different ways (Banihashemi et al., 2021). How threat and deprivation may differentially influence a proximally stress-responsive, central visceral network (PVN, BNST, amygdala and sgACC) is unclear; previous work demonstrated that early experience may shape the sensitivity of these regions to stress (Banihashemi et al., 2011, 2015), however, childhood adversity-related differences in resting-state connectivity specific to this network have not been examined. To this end, the present study examined effects of childhood threat (traumatic events and childhood abuse) and socioeconomic deprivation on the resting-state connectivity of this neural circuit. We hypothesized that threat and deprivation would have differential, potentially opposing effects on resting-state connectivity within this central visceral network.

Overall, we found that childhood threat (namely, early traumatic events, age 0–11) was associated with lower resting-state-connectivity among our central visceral, limbic forebrain-hypothalamic ROIs (BNST-PVN, Amygdala-sgACC and PVN-sgACC). Of these, our most robust findings were that greater exposure to early traumatic events was associated with less PVN-sgACC and amygdala-sgACC connectivity, both of which withstood multiple comparison correction, as well as the addition of adulthood covariates to the model. Contrary to our hypothesis of differential effects of threat and deprivation, we only identified childhood threat as being related to resting-state connectivity within our network, with no significant associations of socioeconomic deprivation (SED) on any ROI-to-ROI connections. Lastly, despite the well-known clinical significance of amygdala-sgACC connectivity, only BNST-PVN connectivity was associated with affective symptoms and disorders, implicating this connection as a potential mediator between childhood threat and affective vulnerability.

### Relationships Between Childhood Threat and Subgenual Anterior Cingulate Cortex-Related Resting-State Connectivity

Two of the three identified ROI-to-ROI relationships with childhood threat involved the sgACC (PVN-sgACC and amygdala-sgACC), a central visceral/visceromotor limbic forebrain region (Vertes, 2004; Alexander et al., 2020) involved in negative affect (Shackman et al., 2011) and emotion regulation, that is also dysregulated in affective disorders (Gotlib et al., 2005; Drevets et al., 2008a,b; Matthews et al., 2009; Alexander et al., 2019). The present study demonstrated a relationship between childhood threat and PVN-sgACC connectivity; this was shown with both early traumatic events (THQ 0-11) and childhood abuse (CTQ Threat), where greater threat was associated with lower PVN-sgACC connectivity. Interestingly, the sgACC has little to no direct projection to the PVN (Öngür et al., 1998; Freedman et al., 2000; Floyd et al., 2001). The sgACC may influence PVN activity via its direct innervation of the BNST (Freedman et al., 2000; Dong et al., 2001a), which sends dense projections to the PVN from its anterolateral and fusiform subnuclei (Figure 1; Dong et al., 2001b; Dong and Swanson, 2004; Maita et al., 2021). Indeed, our findings did indicate that greater exposure to early traumatic events was associated with less BNST-PVN connectivity (discussed further below, Relationship between Childhood Threat and BNST-PVN Resting-State Connectivity).

Our findings also revealed a negative relationship between early traumatic events and amygdala-sgACC resting-state connectivity. The amygdala and sgACC are directly and reciprocally connected (Figure 1); from amygdala to sgACC, projections primarily stem from basal, accessory basal and lateral nuclei (Kim et al., 2018; Sharma et al., 2019). Projections from sgACC to amygdala innervate various subnuclei, including basal, accessory basal, medial and intercalated nuclei (Freedman et al., 2000). Blunt dissection and tractography techniques have also identified putative connections between them (Johansen-Berg et al., 2008; Vergani et al., 2016). To further elaborate the white matter these connections traverse, elegant work by Folloni et al. (2019) demonstrated that in both macaques and humans the amygdalofugal pathway and the uncinate fasciculus extend between the amygdala and sgACC. Our present finding that greater exposure to early traumatic events was associated with less amygdala-sgACC connectivity may indicate microstructural differences in these white matter pathways. Indeed, childhood adversity has been associated with less uncinate fasciculus fractional anisotropy (Eluvathingal et al., 2006; Kumar et al., 2013; Hanson et al., 2015; McCarthy-Jones et al., 2018); however, only a medial bundle from the uncinate extends along the sgACC, while a major section of the amygdalofugal pathway extends along the sgACC (Folloni et al., 2019). Thus, the amygdalofugal pathway may be a promising candidate neural mechanism underlying the relationship between childhood threat and amygdala-sgACC connectivity found here. Further, a recent study showed greater neurite density within the ventral amygdalofugal pathway with age, perhaps indicating greater fiber packing density and/or myelination of the tract (Azad et al., 2021). Future work will be needed to examine childhood adversity-related microstructural differences within this pathway across development and to determine its multimodal relationship to functional connectivity among these ROIs.

Our finding that greater exposure to early traumatic events is associated with less amygdala-sgACC connectivity in a sample of transdiagnostic young adults converges with that of Herringa et al. (2013) who found that greater childhood maltreatment was associated with less amygdala-sgACC connectivity in a late adolescent sample. In younger individuals, however, this relationship may be reversed. Thomason et al. (2015) found that trauma-exposed youth displayed greater centromedial amygdala-sgACC connectivity compared to controls; this work highlights the need for future work examining the connectivity of this central visceral network across development.

The amygdala-sgACC connection has long been thought to be clinically important (Drevets et al., 2008b). Herringa et al. (2013) found that amygdala-sgACC connectivity contributed substantially in mediating the relationship between maltreatment and internalizing symptoms. Depressed adolescents display elevated sgACC-amygdala connectivity (Connolly et al., 2013; Ho et al., 2014) or weaker bottom-up amygdala-sgACC connectivity (Musgrove et al., 2015). Amygdala-sgACC connectivity may also predict treatment response (Taylor et al., 2018; Nakamura et al., 2021). Recent studies highlight the role of amygdala-sgACC connectivity in fear-related encoding, emotional processing/regulation and anxiety (Hakamata et al., 2020; Scharnowski et al., 2020). Hakamata et al. (2020) demonstrated that greater fear encoding strength is associated with greater basolateral amygdala-sgACC connectivity, and that this connectivity was also elevated in anxious participants. Scharnowski et al. (2020) have examined the role of amygdala-sgACC connectivity during automated and effortful emotion regulation; during more automated/less effortful emotion regulation, they found greater amygdala-to-sgACC connectivity. Additionally, they found greater amygdala-to-sgACC modulation among anxious participants during effortful emotion upregulation (Scharnowski et al., 2020). In this context, our amygdala-sgACC findings may suggest less adaptive emotional processing or regulation related to fear-inducing or emotionally salient stimuli, however, such childhood threat-related differences could be indicative of functional impairments that yield vulnerability to affective disorders and/or neuronal adaptations to the early environment that yield resilience (Champagne et al., 2003; Teicher and Samson, 2016; Teicher et al., 2016; Ioannidis et al., 2020).

### Relationship Between Childhood Threat and Bed Nucleus of the Stria Terminalis-Paraventricular Nucleus of the Hypothalamus Resting-State Connectivity

The present study also revealed an association between early traumatic events and BNST-PVN resting-state connectivity; however, this effect was less robust (i.e., did not withstand multiple comparison correction or the addition of adulthood covariates). This relationship did, however, converge with our previous finding that greater childhood threat (both traumatic events and childhood abuse) was associated with less stria terminalis white matter structural integrity (Banihashemi et al., 2021), a white matter bundle that connects these regions (Figure 1; De Olmos and Ingram, 1972; Nieuwenhuys et al., 2008). Taken together, these findings may indicate a reciprocal relationship between BNST-PVN structural and functional connectivity. Further, previous work in rodents shows that ascending noradrenergic/viscerosensory pathways from caudal brainstem collateralize to both BNST and PVN, thus, enabling coordinated modulation of both structures’ response to stress (Banihashemi and Rinaman, 2006). These viscerosensory pathways course through the medial forebrain bundle (Figure 1), which our previous study also revealed may be diminished by childhood threat (Banihashemi et al., 2021). Thus, the medial forebrain bundle may be an indirect pathway underlying the resting-state relationship between BNST and PVN, as well as a neural mechanism underlying the current findings.

The BNST regulates physiological responses to stress not only via its own preautonomic projections but also through its direct connections to the PVN (Maita et al., 2021). Various BNST subnuclei differentially regulate physiological responses to stress (Choi et al., 2007; Crestani et al., 2013). PVN-projecting BNST neurons are primarily GABAergic and recent work has shown that the anteroventral BNST exerts inhibitory influence over HPA responses to stress (Radley et al., 2009; Johnson et al., 2016; Radley and Johnson, 2018) via potential peptidergic mechanisms (Zheng et al., 2019; Povysheva et al., 2021). Considered together, our finding that childhood threat (early traumatic events) was associated with lower BNST-PVN connectivity may indicate the BNST’s diminished capacity to constrain the PVN and stressor-evoked HPA responses, perhaps yielding greater stress reactivity.

It has been proposed that anteroventral BNST-related circuitry is recruited by stress-inducing stimuli, but is uninvolved in tonic HPA regulation (Johnson et al., 2016). Our findings suggest that childhood threat may shape basal BNST-PVN connectivity, however, effects of childhood threat on sgACC-related connectivity (PVN-sgACC and amygdala-sgACC) were more robust, despite BNST-PVN connectivity having closer proximity to the control of stress responses. It is possible that childhood threat may shape the resting-state connectivity of this central visceral network in ways that prime the network to engage differently during stress, with indirect connections (sgACC-related connectivity) more active at rest and direct connections (BNST-PVN) more active during stress.

### Relationship Between Bed Nucleus of the Stria Terminalis-Paraventricular Nucleus of the Hypothalamus Resting-State Connectivity and Affective Disorders

The BNST’s involvement in mediating responses to more distant, less predictable threats implicate it in future-oriented anxiety states, as well as addiction and other psychiatric disorders (Avery et al., 2016; Lebow and Chen, 2016; Clauss, 2019; Clauss et al., 2019; Limbachia et al., 2020). Recent work also indicates stronger BNST-hypothalamus structural connectivity in women, which may underlie sex differences in symptoms related to abstinence from alcohol and risk for relapse (Flook et al., 2021). As the BNST’s anatomical connection to the PVN contributes in part to its ability to respond to threat, our findings may indicate childhood threat-related differences in vulnerability to affective disorders. Indeed, the present study found that greater BNST-PVN resting-state connectivity was associated with less affective symptoms and disorders (i.e., fewer lifetime diagnoses), implicating this connection as a potential mediator between childhood threat and affective vulnerability, although future, larger studies will be necessary to test formal mediation models (Fritz and MacKinnon, 2007).

### Convergence With Large-Scale Networks

The sgACC is considered to be part of the default mode network (DMN), which is involved in self-related mental activity; the DMN is most active when individuals are not engaged in goal-oriented tasks and is deactivated when engaged in cognitive processing (Menon, 2013; Seitzman et al., 2019). The sublenticular extended amygdala and hypothalamus are considered to be part of the salience network (Menon, 2013), although the amygdala may also be considered part of the affective or limbic network (Seitzman et al., 2019). Nevertheless, our findings involved childhood threat-related differences in functional connectivity in cortico-amygdalar-hypothalamic regions that overlap with DMN and salience networks. Expanding literatures indicate that these networks, their nodes and connections between them are altered by childhood adversity (Werff et al., 2012; Marusak et al., 2015; Hoffmann et al., 2018; Cheng et al., 2021; Huang et al., 2021; Rakesh et al., 2021a; Merrick et al., 2018; Silveira et al., 2021), and that these networks are dysregulated in affective disorders (Greicius et al., 2007; Seeley et al., 2007; Jacobs et al., 2014, 2016; Iadipaolo et al., 2018). Thus, our findings may also reflect alterations within these large-scale networks that impact emotion regulation processes (DMN) and orientation to salient internal and external stimuli (salience) (Menon, 2013). Additionally, Kleckner et al. (2017) provided evidence of a large-scale, intrinsic allostatic-interoceptive system and demonstrated that stronger connectivity between hubs within this system supported greater interoceptive ability. This allostatic-interoceptive system is comprised of DMN and salience network regions and includes limbic cortices and subcortical and brainstem visceromotor regions (Kleckner et al., 2017; Ruiz-Rizzo et al., 2020; Sennesh et al., 2022) that converge with our central visceral network of interest (Myers et al., 2005; Banihashemi and Rinaman, 2006; Rinaman et al., 2011; Banihashemi et al., 2015, 2021). Further, Schaan et al. (2019) have shown that childhood maltreatment was associated with less stress-related interoceptive accuracy during a heartbeat perception task. Taken together, our findings indicating lower childhood threat-related central visceral network connectivity may have implications for diminished interoceptive ability and/or accuracy. Future work will be necessary to explicitly examine the neural mechanisms underlying links between childhood adversity and interoceptive ability/capacity.

### Differential Relationships of Childhood Threat and Deprivation on Resting-State Connectivity

Several studies have examined differential relationships between resting-state connectivity and aspects of threat and deprivation dimensions (Cheng et al., 2021; Fadel et al., 2021; Park et al., 2021; Rakesh et al., 2021a). A study examining mesocorticolimbic circuitry in young children found opposing influences of threat and socioeconomic deprivation on ventral tegmental area (VTA)-related resting-state connectivity (Park et al., 2021), with greater threat associated with less VTA-somatomotor connectivity and greater deprivation associated with greater VTA-intraparietal sulcus connectivity. In a large adolescent sample, Rakesh et al. (2021a) found differential effects of threat and deprivation across development: at age 16, greater abuse was associated with less within salience network connectivity, while at age 19, greater neglect was associated with greater within-salience network connectivity, potentially indicating different trajectories for adversity dimensions. Fadel et al. (2021) also found differential relationships of threat and deprivation on salience network connectivity; in a sample of healthy and depressed adults they found opposing relationships of abuse and neglect on within salience network connectivity (i.e., prefrontal cortex-insula), in which greater abuse was associated with greater resting-state connectivity and greater neglect was associated with less resting-state connectivity. The present study did not find effects of socioeconomic deprivation, as defined by maximum parental education level (reverse coded), on any ROI-to-ROI connection. Effects of CTQ Deprivation (neglect) on central visceral network connectivity were also explored and no significant relationships were found (data not shown). It is possible that socioeconomic deprivation will have a greater impact on stressor-evoked activity and connectivity within this central visceral network than on its resting-state connectivity. Future work on this network will be needed to investigate different aspects of the deprivation construct (e.g., neighborhood and cognitive deprivation).

### Limitations and Future Directions

A limitation of this study is its cross-sectional design examining young adults; however, participants were specifically recruited across a continuum of physical abuse severity, with individuals across a spectrum of affective symptom severity including those with depression, anxiety and trauma-related disorders. This recruitment strategy achieved a relatively even distribution across childhood socioeconomic status, as well. Nevertheless, future prospective work will be needed to examine distinct dimensions of childhood adversity and how they differentially impact this central visceral network across development. Additionally, future studies designed to be statistically powered for detecting realistic effect sizes for mediation are necessary to further examine central visceral network components as mediators of the relationship between childhood adversity and affective symptoms/disorders.

Regions of interest in the present study were defined using a template for manual segmentation [sgACC, BNST and PVN (Banihashemi et al., 2015; Wu et al., 2019)]. Greater accuracy and precision are necessary to define specific subnuclei within these ROIs and to examine additional components of the network (e.g., brainstem nuclei), which may benefit from high-field acquisitions. Improvements in manual segmentation approaches for these regions and continued advancements in automated segmentations would also benefit the examination of these brain regions, particularly in humans. Future work will be needed to capitalize on current advances (Avery et al., 2014; Saygin et al., 2017; Wolff et al., 2018).

## Summary and Conclusion

This study provides novel evidence that childhood threat may influence a central visceral network. Analyses revealed that childhood threat is associated with lower connectivity among our ROIs (PVN-sgACC, amygdala-sgACC and BNST-PVN). These findings have functional and clinical implications that suggest potential alterations in emotion regulation and processing, orienting responses to salient stimuli, and stress and threat reactivity. Further, our results demonstrate that BNST-PVN connectivity may provide a novel link between childhood threat and affective symptoms and disorders. In conclusion, exposure to threat during early development may entrain altered patterns of resting-state connectivity between these stress-related regions in ways that contribute to dysregulated neural and physiological responses to stress and subsequent affective psychopathology. Investigating how this network links childhood adversity and affective symptoms may elucidate underlying neural mechanisms of affective disorders, as well as guide interventions targeting these brain structures.

## Data Availability Statement

The datasets presented in this article are not readily available because they are still undergoing primary analyses. The data that support the findings of this study will be made available from the corresponding author upon request in the future. Requests to access the datasets should be directed to LB, layla.banihashemi@pitt.edu.

## Ethics Statement

The studies involving human participants were reviewed and approved by the University of Pittsburgh Institutional Review Board. The patients/participants provided their written informed consent to participate in this study.

## Author Contributions

LB conceptualized the research, acquired funding, conducted the research, performed formal analysis, wrote the original draft, and reviewed and edited the manuscript. CP conducted the research, managed and coordinated the research, performed formal analysis, and wrote portions of the Methods. AR performed formal analysis and implemented code and algorithms. HK provided programming, implemented code and algorithms, and reviewed and edited the manuscript. MW contributed to creation of models and reviewed and edited the manuscript. BS wrote portions of the Discussion and reviewed and edited the manuscript. JS wrote portions of the Introduction. MS provided data curation. AG provided mentorship, oversight, and resources. HA provided mentorship, oversight, and resources and reviewed and edited the manuscript. All authors contributed to the article and approved the submitted version.

## Conflict of Interest

MW is a statistical consultant for Noctem, unrelated to this work. AG serves as CEO and holds equity in Rehat, LLC and has also served as a consultant for Jazz Pharmaceuticals, Inc., unrelated to this work. The remaining authors declare that the research was conducted in the absence of any commercial or financial relationships that could be construed as a potential conflict of interest.

## Publisher’s Note

All claims expressed in this article are solely those of the authors and do not necessarily represent those of their affiliated organizations, or those of the publisher, the editors and the reviewers. Any product that may be evaluated in this article, or claim that may be made by its manufacturer, is not guaranteed or endorsed by the publisher.

## Footnotes

1
http://www.fil.ion.ucl.ac.uk/spm/
