# Supplementary material for: Childhood Threat Is Associated With Lower Resting-State Connectivity Within a Central Visceral Network
Source: Front Psychol. 2022 Mar 3;13:805049. doi: 10.3389/fpsyg.2022.805049 (PMC8927539; doi:10.3389/fpsyg.2022.805049)
Supplement: Supplementary file 1 [file Data_Sheet_1.pdf]

Supplementary Material

Supplementary Figures and Tables

Table S1. Participant Characteristics (n=100)

Characteristic	Mean	S.D.	Range
Age (years)	27.28	3.99	21-35
CTQ Threat (abuse)	31.25	13.59	15-69
CTQ Deprivation (neglect)	21.14	8.79	10-46
CTQ total score	52.39	21.11	25-100
THQ (age 0-11)	2.21	2.46	0-13
THQ (age 12-17)	2.70	2.57	0-11
Parental education level	5.29	2.04	1-8
Education level	5.19	1.56	1-8
BDI II total score	11.64	10.79	0-49
PCL-C total	32.35	13.92	17-75
Perceived stress	16.97	8.75	2-37
STAI-T	41.76	13.33	20-77
NEO: Neuroticism (%)	36.86	10.18	13-59
NEO: Extraversion (%)	39.54	7.96	12-56
NEO: Openness (%)	33.58	4.73	19-42
NEO: Agreeableness (%)	44.10	4.96	28-55
NEO: Conscientiousness (%)	43.88	7.42	20-59

CTQ = Childhood Trauma Questionnaire; THQ = Trauma History Questionnaire, Education Level = 0 - No high school diploma, 1 – GED, 2 – High school diploma, 3 – Technical training, 4 – Some college, no degree, 5 – Associate degree, 6 – Bachelor's degree, 7 – Master's degree, 8 – MD/PhD/JD/PharmD; BDI = Beck Depression Inventory; PCL-C = PTSD Checklist – Civilian Version; STAI-T = State-Trait Anxiety Inventory-Trait; NEO = NEO Personality Inventory

RESULTS

Childhood Threat, Deprivation and Central Visceral Network Resting-state Connectivity

Table S2. Early Traumatic Events (THQ, age 0-11): Examination of Potential Outliers

THQ 0-11 Value	Mahalanobis's distance	Critical Value 5 IVs	Critical Value 8 IVs
13	26.77*#	20.52	26.13
12	21.93*	20.52	26.13

*Exceeds critical value for 5 Independent Variables (IVs); # Exceeds critical value for 8 IVs.

Table S3. Abbreviated Hierarchical Linear Regression Results without THQ 0-11 = 13

		**BNST-PVN**			**Amygdala-sgACC**			**PVN-sgACC**		
Step	Variable	St. Beta	t	*p*	St. Beta	t	*p*	St. Beta	t	*p*
2	**THQ 0-11**	-.143	-1.363	.176	-.277	-2.735	**.007***	-.279	-2.895	**.005***
3	**THQ 0-11**	-.067	-.581	.562	-.291	-2.556	**.012**	-.250	-2.322	**.022**

Bold values indicate significance at p<0.05; an asterisk indicates survival of FDR correction (0.05) for six tests [Amygdala-sgACC (adjusted *p* = 0.021); PVN-sgACC (adjusted *p* = 0.030)].

Table S4. Abbreviated Hierarchical Linear Regression Results without THQ 0-11 = 13 & 12

		**BNST-PVN**			**Amygdala-sgACC**			**PVN-sgACC**		
Step	Variable	St. Beta	t	*p*	St. Beta	t	*p*	St. Beta	t	*p*
2	**THQ 0-11**	-.115	-1.081	.282	-.207	-1.979	.051	-.285	-2.915	**.004***
3	**THQ 0-11**	-.060	-.536	.593	-.223	-2.000	**.049**	-.258	-2.493	**.015**

Bold values indicate significance at p<0.05; an asterisk indicates survival of FDR correction (0.05) for six tests [PVN-sgACC (adjusted *p* = 0.024)].

Table S5. Hierarchical Linear Regression Results: Childhood Threat (CTQ Physical Abuse) and Central Visceral Network Resting-State Connectivity

		**PVN-sgACC**		
Step	Variable	St. Beta	t	*p*
1	Age	.113	1.166	.247
	Race	-.038	-.388	.699
	Sex	.301	3.111	**.002**
2	Age	.139	1.386	.169
	Race	.020	.206	.837
	Sex	.264	2.703	**.008**
	**CTQ Physical Abuse**	-.255	-2.309	**.023***
	Socioeconomic Deprivation	.113	1.040	.301
3	Age	.173	1.517	.133
	Race	.015	.149	.882
	Sex	.223	2.158	**.034**
	**CTQ Physical Abuse**	-.212	-1.805	.074
	Socioeconomic Deprivation	.126	1.063	.290
	THQ >18	-.006	-.051	.959
	Adulthood SES	-.018	-.175	.861
	Negative Life Events	-.149	-1.267	.208

Bold values indicate significance at $p<0.05$; an asterisk indicates survival of FDR correction (0.05) for three tests.

Table S6. Regression Results: Childhood Threat (CTQ Sexual Abuse) and Central Visceral Network Resting-State Connectivity

		PVN-sgACC		
Step	Variable	St. Beta	t	*p*
1	Age	.113	1.166	.247
	Race	-.038	-.388	.699
	Sex	.301	3.111	**.002**
2	Age	.142	1.428	.156
	Race	-.013	-.134	.894
	Sex	.292	3.054	**.003**
	**CTQ Sexual Abuse**	-.250	-2.513	**.014***
	Socioeconomic Deprivation	.074	.728	.469
3	Age	.193	1.714	.090
	Race	-.010	-.100	.921
	Sex	.228	2.247	**.027**
	**CTQ Sexual Abuse**	-.248	-2.480	**.015***
	Socioeconomic Deprivation	.108	.971	.334
	THQ >18	.001	.006	.995
	Adulthood SES	-.039	-.380	.705
	Negative Life Events	-.205	-1.815	.073

Bold values indicate significance at $p<0.05$; an asterisk indicates survival of FDR correction (0.05) for three tests.

Later Traumatic Events (THQ, age 12-17)

Six resting-state ROI-to-ROI connections were examined (Amygdala-BNST, Amygdala-PVN, Amygdala-sgACC, BNST-PVN, BNST-sgACC and PVN-sgACC). Of these, THQ 12-17 had a significant, negative association with Amygdala-sgACC (ß = -0.218; p = 0.044) and PVN-sgACC connectivity (ß = -0.220; p = 0.034). Neither relationship survived multiple comparison correction; neither relationship remained significant when adulthood trauma (age >18), adulthood SES and negative life events were added to the model, Table S7). Socioeconomic deprivation (SED, maximum parental education level reverse coded) did not have a significant effect on any ROI-to-ROI connection examined.

Table S7. Hierarchical Linear Regression Results: Childhood Threat (Later Traumatic Events, age 12-17) and Central Visceral Network Resting-State Connectivity

		**Amygdala-sgACC**			**PVN-sgACC**		
Step	Variable	St. Beta	t	*p*	St. Beta	t	*p*
1	Age	-.036	-.358	.721	.113	1.166	.247
	Race	-.177	-1.750	.083	-.038	-.388	.699
	Sex	.007	.068	.946	.301	3.111	**.002**
2	Age	-.056	-.540	.590	.111	1.111	.269
	Race	-.145	-1.436	.154	-.009	-.093	.926
	Sex	.042	.416	.678	.327	3.370	**.001**
	**THQ 12-17**	-.218	-2.041	**.044**	-.220	-2.154	**.034**
	Socioeconomic Deprivation	.153	1.374	.173	.090	.849	.398
3	Age	-.025	-.203	.840	.159	1.379	.171
	Race	-.147	-1.423	.158	-.016	-.165	.869
	Sex	.044	.392	.696	.284	2.656	**.009**
	**THQ 12-17**	-.225	-1.880	.063	-.187	-1.646	.103
	Socioeconomic Deprivation	.144	1.211	.229	.085	.747	.457
	THQ >18	-.067	-.528	.599	-.028	-.233	.816
	Adulthood SES	-.060	-.539	.591	-.080	-.764	.447
	Negative Life Events	.015	.117	.907	-.123	-1.003	.319

Bold values indicate significance at p<0.05; THQ 12-17 effects did not survive FDR correction (0.05) for six tests.

Table S8. Relationships between Childhood Adversity and Affective Symptoms (n=100).

		CTQ Threat	THQ 0-11	THQ 12-17	CTQ Deprivation	SED	BDI-II	PCL-C	Lifetime Diagnoses
**CTQ Threat**	r	--							
	p								
**THQ 0-11**	r	.535**	--						
	p	.000							
**THQ 12-17**	r	.580**	.539**	--					
	p	.000	.000						
**CTQ Deprivation**	r	.769**	.462**	.534**	--				
	p	.000	.000	.000					
**Socioeconomic Deprivation (SED)**	r	.411**	.175	.337**	.404**	--			
	p	.000	.081	.001	.000				
**BDI-II**	r	.511**	.331**	.322**	.489**	.340**	--		
	p	.000	.001	.001	.000	.001			
**PCL-C**	r	.556**	.463**	.345**	.544**	.300**	.850**	--	
	p	.000	.000	.000	.000	.002	.000		
**Lifetime diagnoses**	r	.637**	.484**	.392**	.621**	.378**	.672**	.696**	--
	p	.000	.000	.000	.000	.000	.000	.000	

**. Pearson correlation is significant at the 0.01 level (2-tailed).
